# Burden of Non-Communicable Diseases and Emerging Attention in Gandaki Province, Nepal

**DOI:** 10.31729/jnma.8922

**Published:** 2025-03-31

**Authors:** Khim Bahadur Khadka, Deepak Paudel

**Affiliations:** 1 Ministry of Health, Gandaki Province, Pokhara Kaski, Nepal; 2 Center for International Health, Ludwig Maximilians University, Munich, Germany

**Keywords:** *Gandaki*, *mass screening*, *non-communicable disease*

## Abstract

Non-communicable diseases have become a major health challenge globally, including in Nepal. Deaths due to non-communicable diseases in Nepal are increasing, accounting more than half of total deaths. Major contributors to deaths due to non-communicable diseases include cardiovascular diseases, chronic respiratory diseases, cancers, digestive diseases such as pancreatitis and cirrhosis of liver, and diabetes. To address this, Gandaki Province organized integrated screening sessions for non-communicable diseases covering 2,976 high-risk individuals aged 40 years and above from six districts. The preliminary findings of screenings revealed a high prevalence of hypertension, high blood sugar, obesity, and abnormal cholesterol levels. Designating Falgun as the month for the non-communicable diseases screening campaign throughout the country helps in promoting awareness, screening, and management of these diseases through effective coordination and resource allocation for successful impact.

## INTRODUCTION

Non-communicable diseases (NCDs) have emerged as a significant health challenge globally^1^, including in Nepal and specifically Gandaki Province. Between 1990 and 2021, deaths from NCDs in Nepal increased from 360 to 438 per 100,000 people.^2^ In 2021 alone, NCDs caused 136,225 deaths and resulted in 19,601 disability-adjusted life years (DALYs) per 100,000 population in Nepal. Major NCDs responsible for these deaths include cardiovascular diseases (55,697 deaths), chronic respiratory diseases (34,347 deaths), neoplasms (15,832 deaths), digestive diseases such as pancreatitis and cirrhosis of liver (11,428 deaths), and diabetes (6,549 deaths) and NCDs account for 53.87% of deaths in Nepal.^2^ While sub-national Global Burden of Disease (GBD) data is unavailable, anecdotal evidence and medical records from healthcare providers in Gandaki Province indicate an increasing prevalence of NCDs.

## COMMNITY BASED MASS SCREENING

In response to this growing concern, Gandaki Province organized 11 events of two-day integrated screening, management, and counselling sessions across various locations, utilizing a multidisciplinary team approach.

These sessions aimed to identify and facilitate timely management of NCDs. The integrated NCD screening sessions were strategically conducted in diverse locations: three in Galkot, Baglung; one in Amalachaur, Baglung; three in Pokhara, Kaski; one in Putalibazar, Syangja; one in Madhyabindu, Nawalparasi; one in Ghiring, Tanahu; and one in Myagdi, Beni. These sessions took place from Jestha 15, 2081 to Falgun 1, 2081 (May 28, 2024 to February 13, 2025).

Nearly 40 health workers and volunteers were mobilized for each session, collectively assessing 2,976 high-risk individuals with a mean age of 54 years. Each session attracted an average of 250-300 clients. Assessments included blood tests for random sugar, triglycerides, cholesterol, high-density lipoprotein (HDL) and low-density lipoprotein (LDL), serum glutamic-oxaloacetic transaminase (SGOT) for liver function, and urine tests for albumin, creatinine, and sugar. Participants were also evaluated for body mass index (BMI) using anthropometry (height, weight) data, systolic and diastolic blood pressure, anxiety and depression using internationally validated General Anxiety Disorder-7 and depression using Patient Health Questionnaire-9 tools.^3^

The preliminary analysis of screening data revealed an alarming prevalence of NCDs and associated risk factors in Gandaki Province. Among those screened, 23.60% had stage I hypertension, 14.90% had stage II hypertension, and 25.50% were in the pre-hypertensive stage. Although these findings are from a relatively high-risk population, they are concerning for public health professionals and policymakers. Additionally, 11.10% had random blood sugar levels exceeding 140 mg/dl, and 11.70% had fasting blood sugar levels exceeding 110 mg/dl.

When assessed for body mass, 10.90% were obese (BMI >25kg/m^2^), and 32.00% were overweight (BMI >23kg/m^2^). Elevated creatinine levels (>1.5mg/dl) were found in 1.40% of participants, SGOT levels were higher (>35 U/L) in 27.70% of participants, and total cholesterol levels were elevated (>200 mg/dl) in 52.20% of participants. Similarly, LDL levels were higher (>160 mg/dl) in 2.6% and HDL levels were lower (<40 mg/dl) in 13.40% of participants. Additionally, 1.80% of participants had traces of protein in their urine, and 3.40% had sugar in their urine. When assessed for anxiety and depression, 6.90% had moderate anxiety, 4.40% had severe anxiety, 4.80% had moderate depression, and 3.50% had moderate to severe or severe depression. Among the participants, 15.40% had a history of smoking, and 8.85% were current smokers.

## PUBLIC HEALTH PRIORITY AND CALL FOR ACTION

These findings highlight an urgent need to raise awareness about NCD prevention and to enhance timely screening and management of these health issues. Public health programs should be designed, implemented, and followed up in collaboration with local, provincial, and federal governments. In light of the NCD burden, the Government of Nepal has designated Falgun (mid-February to mid-March) as the month of the NCD campaign.^4^ This campaign focuses on targeted screening for diabetes, hypertension, kidney diseases, and BMI, and aims to raise awareness about weight management, the harmful effects of smoking, alcohol use, and excessive salt intake.

The federal Minister of Health and Population, Mr. Pradip Paudel, inaugurated the one-month campaign in Pokhara, Gandaki Province, and called for the support of all stakeholders. Individuals with identified health issues in these sessions are referred and linked to health systems and the Package of Essential Non-Communicable Diseases (PEN). The program is being disseminated through various channels, including television, radio, local newspapers, social media, and interpersonal contacts during meetings and other interactions, which are proven to improve uptake of the screening services.^5^

Raising awareness about the escalating trend of NCDs is imperative, emphasizing individual responsibility for adopting healthy behaviors and undergoing timely screenings, especially among high-risk groups like those over 40 years of age. The ongoing campaign has successfully captured the attention of the public, policymakers, and researchers, underlining the significance of mass screening interventions.

Enhancing the availability and utilization of NCD screening services necessitates the provision of laboratory investigations, medications, equipment, and healthcare providers, along with fostering a strong patient-provider relationship for sustainable health outcomes.^6^ Effective intersectoral and intergovernmental coordination is essential to ensure the success of these campaigns in the timely identification and management of NCDs, ultimately reducing their burden on the population.
